# Reduced-order state-space reformulation for task-space predictive control of an under-actuated hydraulic boom

**DOI:** 10.3389/frobt.2026.1853243

**Published:** 2026-08-06

**Authors:** Elham Kowsari, Reza Ghabcheloo

**Affiliations:** Faculty of Engineering and Natural Sciences, Tampere University, Tampere, Finland

**Keywords:** hydraulic boom control, nonlinear model predictive control, state-space reformulation, sway reduction, task-space control, underactuated systems

## Abstract

Hydraulic booms used in material handling are typically commanded in cylindrical task-space coordinates, while dynamic models used for control are commonly formulated in joint coordinates. This mismatch leads to unnecessarily high-dimensional system representations when predictive control is applied to underactuated suspended loads. This paper addresses this modeling inconsistency by introducing a reduced-order state-space reformulation of grapple sway dynamics directly in cylindrical task-space velocity coordinates. The proposed input-consistent state transformation eliminates the dependence of the dynamics on input accelerations by embedding actuator–sway coupling within modified velocity states, yielding a nonlinear model driven solely by cylindrical velocity commands. Based on this representation, a nonlinear model predictive control (NMPC) framework is developed for simultaneous goal reaching, sway suppression, and obstacle avoidance. The NMPC cost function is designed to capture the practical trade-off between rapid boom motion and suppression of suspended load oscillations. In addition, approximate hydraulic actuator dynamics identified from a high-fidelity AMESim simulator model of a forwarder crane are incorporated to better reflect realistic boom behavior. Simulation studies in representative boom operation scenarios demonstrate that the proposed formulation enables effective sway regulation and accurate task-space motion control using a reduced set of states without requiring full joint-space models or payload sensing. The results indicate that the proposed cylindrical state-space reformulation simplifies predictive control design for underactuated hydraulic booms while preserving the essential dynamics of the suspended load.

## 1 Introduction

Hydraulic booms are widely used in construction, forestry, and material-handling applications to manipulate heavy loads in complex environments. Many such systems employ suspended tools such as grapples, whose motion introduces underactuated sway dynamics that degrade positioning accuracy and operational safety Schubert et al. (2019); Zhai et al. (2024). Effective control of boom motion therefore requires simultaneous regulation of the boom-tip trajectory and suppression of load oscillations.

Hydraulic boom manipulation remains challenging due to nonlinear dynamics, actuator limitations, and environmental constraints. In practice, oscillations of the suspended load can significantly reduce positioning precision and increase the risk of collisions with nearby structures, particularly in confined construction and forestry environments Ecker et al. (2025); Gao et al. (2025); Dvořák et al. (2008). These factors complicate efforts toward automation and precise motion control.

Accurate modeling of the coupled boom–load dynamics is essential for effective control. In many forestry and construction boom systems, motion commands are issued in cylindrical task-space coordinates corresponding to slewing, radial extension, and vertical lifting. However, most dynamic models used for control of hydraulic booms are formulated either in joint coordinates or in Cartesian coordinates derived from full boom kinematics Kalmari et al. (2017). While these representations are suitable for analysis, they do not directly correspond to the control interface commonly used in practical machines.

In many sway models of suspended loads, the dynamics are expressed in terms of boom-tip accelerations Esim et al. (2025). When cylindrical task-space commands are substituted into these acceleration expressions, second derivatives of the commanded velocities appear in the dynamic equations. Consequently, conventional state definitions lead to models that depend on derivatives of the control inputs, preventing the system from being written in the standard nonlinear state-space form required for predictive control Ambrosino et al. (2021).

Recent studies have addressed sway reduction and motion control of hydraulic booms using predictive and model-based approaches. Nonlinear predictive control for forestry cranes is typically formulated in joint coordinates assuming availability of full crane kinematics Kalmari (2015). Flatness-based stabilization methods for knuckle-boom cranes similarly rely on inversion of the joint-space model and payload position sensing Schubert et al. (2019). Trajectory generation approaches for sway damping also employ either joint-space states or Cartesian tip motion derived from joint kinematics Jebellat and Sharf (2023).

Despite differences in control and planning strategies, most existing approaches formulate crane dynamics in joint or Cartesian coordinates derived from full boom kinematics. Under this representation, the underactuated sway motion is only indirectly influenced by actuator commands through the joint model, which leads to high-dimensional representations that are not aligned with the cylindrical task-space velocity commands used to operate hydraulic booms in practice.

In contrast, this work reformulates the grapple sway dynamics directly in cylindrical task-space velocity coordinates using an input-consistent state transformation that eliminates the dependence on input accelerations while preserving the coupling between boom motion and sway dynamics. The resulting formulation yields a reduced-order nonlinear state-space model driven by cylindrical velocity inputs, enabling predictive control design using a lower-dimensional model consistent with the practical control interface of hydraulic boom systems.

The remainder of the paper is organized as follows. Section 2 reviews related work on modeling and control of hydraulic cranes and suspended load systems. Section 3 defines the control problem and summarizes the main contributions. Section 4 presents the reduced-order state-space model of the boom–grapple system. Section 5 introduces the proposed NMPC formulation and constraint handling. Section 6 incorporates actuator dynamics into the prediction model. Section 7 discusses the analytical NMPC problem-size comparison and the modeling assumptions underlying the proposed formulation. Section 8 presents the simulation scenarios and results. Finally, Section 9 concludes the paper and discusses directions for future work.

## 2 Related work

Classical dynamic models of hydraulic booms used in control and planning literature are typically expressed either in joint coordinates or in Cartesian tip coordinates derived from boom kinematics. For example, nonlinear model predictive control (NMPC) approaches for forestry cranes formulate the system dynamics directly in joint coordinates, assuming availability of joint states and full crane kinematics Kalmari (2015). Flatness-based payload stabilization methods for knuckle-boom cranes similarly rely on inversion of the joint-space model and require payload position sensing Schubert et al. (2019). Trajectory generation approaches for sway damping also adopt joint-space states or Cartesian tip motion variables as the planning coordinates Jebellat and Sharf (2023).

Although these approaches differ in control strategy and planning framework, they share a common modeling perspective: the crane dynamics are formulated using joint coordinates or Cartesian tip variables derived from the boom kinematics. Consequently, the sway dynamics are only indirectly influenced by actuator commands through the joint-space model.

In practical hydraulic boom operation, however, motion is typically commanded through cylindrical task-space velocity inputs corresponding to slewing, radial extension, and vertical lifting. This creates a mismatch between the control interface used in real machines and the modeling coordinates adopted in many existing approaches.

Although the above methods provide important tools for crane motion control, sway reduction, and trajectory generation, their modeling choices introduce limitations for the NMPC formulation considered in this work. Joint-space formulations require full crane kinematics and higher-dimensional joint-state representations. Cartesian tip formulations reduce dependence on individual joint coordinates, but they still describe the system through tip-position and tip-velocity variables and typically require acceleration-level inputs. In contrast, practical hydraulic forwarder booms are commonly commanded through cylindrical task-space velocity inputs corresponding to slewing, radial motion, and vertical motion. This creates a gap between the command variables available at the machine interface and the coordinates used in many prediction models. The present work addresses this gap by deriving an input-consistent reduced-order state-space model directly driven by cylindrical task-space velocity commands.

Table 1 summarizes representative modeling choices in crane control and planning literature and compares them with the formulation proposed in this work. Existing approaches typically rely on joint coordinates or Cartesian tip variables and require higher-dimensional state representations. In contrast, the formulation developed in this paper directly adopts cylindrical task-space velocity inputs and introduces a reduced-order state-space representation that captures the coupling between actuator motion and sway dynamics while avoiding explicit dependence on joint-space states.

**TABLE 1:** Comparison of state and input representations in representative boom control and planning approaches.

Approach	State vector	Inputs	# states
Kalmari Kalmar (2015)	$\left[\theta_{1},\theta_{2},\theta_{3},d_{4},\theta_{5},\theta_{6},{\dot{\theta }}_{1},{\dot{\theta }}_{2},{\dot{\theta }}_{3},{\dot{d}}_{4},{\dot{\theta }}_{5},{\dot{\theta }}_{6}\right]$	$\left[{\ddot{\theta }}_{1},{\ddot{\theta }}_{2},{\ddot{\theta }}_{3},{\ddot{d}}_{4}\right]$	12
Jebellat (ICRA) Jebellat and Sharf (2023)	$\left[\theta_{1},\theta_{2},\theta_{3},d_{4},{\dot{\theta }}_{1},{\dot{\theta }}_{2},{\dot{\theta }}_{3},{\dot{d}}_{4},\theta_{5},{\dot{\theta }}_{5},\theta_{6},{\dot{\theta }}_{6}\right]$	$\left[{\ddot{\theta }}_{1},{\ddot{\theta }}_{2},{\ddot{\theta }}_{3},{\ddot{d}}_{4}\right]$	12
Jebellat (T-ASE) Jebellat and Sharf (2023)	$\left[x_{\mathrm{tip}},y_{\mathrm{tip}},z_{\mathrm{tip}},{\dot{x}}_{\mathrm{tip}},{\dot{y}}_{\mathrm{tip}},{\dot{z}}_{\mathrm{tip}},\theta_{5},{\dot{\theta }}_{5},\theta_{6},{\dot{\theta }}_{6}\right]$	$\left[{\ddot{x}}_{\mathrm{tip}},{\ddot{y}}_{\mathrm{tip}},{\ddot{z}}_{\mathrm{tip}}\right]$	10
Schubert Schubert et al. (2019)	$\left[x_{\mathrm{tip}},y_{\mathrm{tip}},z_{\mathrm{tip}},{\dot{x}}_{\mathrm{tip}},{\dot{y}}_{\mathrm{tip}},{\dot{z}}_{\mathrm{tip}},\theta_{5},{\dot{\theta }}_{5},\theta_{6},{\dot{\theta }}_{6}\right]$	Flatness-based input	10
Proposed	$\left[\theta_{5},{\dot{\theta }}_{5}-\alpha_{5}{\dot{\theta }}_{1}-\alpha_{6}\dot{r}-\alpha_{7}\dot{z},\theta_{6},{\dot{\theta }}_{6}-\beta_{5}{\dot{\theta }}_{1}-\beta_{6}\dot{r}-\beta_{7}\dot{z},\theta_{1},r,z\right]$	$\left[{\dot{\theta }}_{1},\dot{r},\dot{z}\right]$	7

The practical relevance of the reduced-order formulation is related to the command structure of hydraulic forwarder booms. In industrial forwarder machines, boom motion is commonly commanded through cylindrical task-space velocity inputs, namely, slewing velocity, radial velocity, and vertical velocity. Therefore, higher-dimensional joint-space or Cartesian acceleration-based formulations introduce a mismatch between the prediction model and the signals available at the machine-level control interface. The proposed reformulation removes this mismatch by deriving a compact state-space model directly driven by cylindrical velocity commands. Consequently, the dimensionality reduction reduces the NMPC problem size while also simplifying implementation, since the optimizer operates on the same type of inputs that are available in the practical hydraulic control system.

The comparison in Table 1 should be interpreted as a formulation-level comparison rather than a complete closed-loop performance benchmark. Existing joint-space, Cartesian-tip, and anti-sway control approaches are based on different modeling assumptions, input definitions, sensing requirements, and control objectives. A direct numerical benchmark would require re-implementing each method with identical actuator dynamics, constraints, obstacle configurations, solver settings, and measurement assumptions. This is outside the scope of the present work. The contribution of this paper is therefore not a claim of universal superiority over all existing controllers, but the development of a compact input-consistent prediction model that is directly compatible with cylindrical velocity commands used in practical hydraulic boom systems.

## 3 Problem definition and contributions

The primary challenge addressed in this work is the precise and efficient motion of a hydraulic boom equipped with a suspended grapple from an initial position to a desired target location while avoiding obstacles along the trajectory. This problem frequently arises in construction and material-handling applications where cranes must operate in cluttered environments while maintaining motion accuracy and operational safety.

The presence of a suspended load introduces significant dynamic challenges. The grapple behaves as an underactuated pendulum system that exhibits oscillatory sway during boom motion. Rapid boom movements may excite these oscillations, whereas aggressive sway suppression can slow down task execution. Achieving both efficient motion and stable load behavior therefore requires a control strategy capable of balancing these competing objectives.

Another important challenge arises from the mismatch between the practical control interface of hydraulic booms and the modeling coordinates commonly used in control design. In practice, boom motion is commanded using cylindrical task-space velocities corresponding to slewing, radial extension, and vertical motion. However, many control-oriented models are formulated in joint coordinates or Cartesian tip coordinates, which can lead to higher-dimensional or less directly implementable prediction models for NMPC.

Although existing methods provide important tools for crane motion control, sway reduction, and trajectory generation, their modeling choices introduce limitations for the NMPC formulation considered in this work. Joint-space formulations require full crane kinematics and joint-state representations, while Cartesian tip-coordinate formulations typically describe the system through tip-position, tip-velocity, or acceleration-level variables. This creates a gap between the command variables available at the machine interface and the coordinates used in many existing prediction models.

The present work also differs from our previous study on sway reduction of forestry cranes. The previous work focused on optimal sway reduction using an open-loop/feedforward inverse-dynamics approach. In that study, the main objective was to shape the boom control command by reducing frequency components that excite oscillatory motion of the grapple. The method was evaluated in a high-fidelity simulation environment and was mainly concerned with sway reduction for a given motion command.

In contrast, the present paper focuses on the modeling and NMPC formulation itself. It derives a reduced-order, input-consistent nonlinear state-space model directly in cylindrical task-space velocity coordinates. The proposed transformation removes the explicit dependence on input accelerations and makes the model suitable as an NMPC prediction model driven by the available machine-level velocity commands.

To address this gap, this paper proposes a predictive control framework based on an input-consistent reduced-order state-space representation of the boom–grapple system. The main contributions of this work are summarized as follows:

- A reduced-order state-space reformulation of the grapple sway dynamics is derived directly in cylindrical task-space velocity coordinates, consistent with the practical command interface of hydraulic boom systems.
- An input-consistent state transformation is introduced to eliminate the explicit dependence on input accelerations while preserving the coupling between boom motion and grapple sway dynamics.
- The resulting nonlinear state-space model is used as an NMPC prediction model for simultaneous goal reaching, sway suppression, obstacle avoidance, and actuator-dynamics-aware control.
- Actuator dynamics identified from a high-fidelity industrial forwarder-crane simulator are integrated into the NMPC framework to improve the practical relevance of the prediction model.

## 4 Reduced-order state-space model of grapple sway dynamics

This section presents the dynamic model used to describe the sway motion of the grapple attached to the boom tip. The goal is to derive a state-space representation compatible with cylindrical task-space velocity commands, which correspond to the practical control interface of hydraulic booms.

We begin by deriving the sway dynamics of the suspended grapple using a simplified double-pendulum representation. These equations describe the coupling between boom–tip motion and oscillations of the suspended load. However, when expressed using conventional generalized coordinates, the resulting model depends on accelerations of the boom–tip motion.

To obtain a model suitable for predictive control, the sway dynamics are reformulated through an input-consistent state transformation that eliminates the dependence on input accelerations. The resulting representation yields a reduced-order nonlinear state-space model driven directly by cylindrical velocity inputs.

### 4.1 Grapple sway dynamics

**FIGURE 1:**
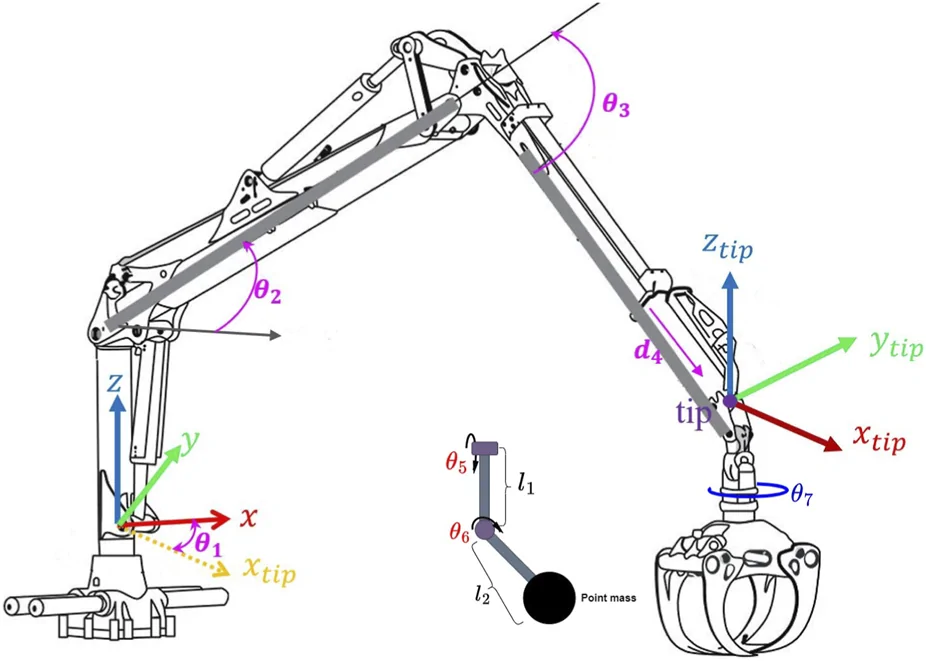
Kinematic structure of the hydraulic boom with suspended grapple and coordinate definitions used for modeling.

The sway motion of the grapple is governed by the passive joints that connect the suspended load to the boom tip. As shown in Figure 1, the suspended system is characterized by two sway angles, $\theta_{5}$ and $\theta_{6}$ , describing the angular motion of the grapple relative to the boom tip. The rotation $\theta_{7}$ , actuated by the hydraulic motor, is not included in the sway model because it does not govern the free oscillatory motion considered in this work.

To capture the coupling between boom motion and suspended-load oscillations, the grapple is modeled as a two-degree-of-freedom pendulum system attached to the boom tip. The model includes gravitational effects, viscous damping at the passive joints, and excitation due to boom-tip accelerations. Let $l_{1}$ denote the distance between the first and second passive joints, and let $l_{2}$ denote the distance from the second passive joint to the center of mass of the grapple.

Using the Lagrangian formulation with generalized coordinates $q={\left[\theta_{5},\theta_{6}\right]}^{T}$ , the sway dynamics can be written in terms of the boom–tip accelerations ${\ddot{x}}_{\mathrm{tip}}$ , ${\ddot{y}}_{\mathrm{tip}}$ , and ${\ddot{z}}_{\mathrm{tip}}$ as shown in Equations 1, 2:

$$\begin{array}{cc} {\ddot{\theta }}_{5} & =\frac{1}{m_{0}l_{0}^{2}+ml_{3}^{2}}\left[-\left(m_{0}l_{0}+ml_{3}\right)\left(\cos\theta_{5}\;\ddot{y}tip+\sin\theta_{5}\;{\ddot{z}}_{tip}\right)\right.\\ & \left.-b_{5}{\dot{\theta }}_{5}\left(m_{0}l_{0}+ml_{3}\right)g\sin\theta_{5}+2\;ml_{2}l_{3}\sin\theta_{6}{\dot{\theta }}_{6}{\dot{\theta }}_{5}\right] \end{array}$$ (1)

$$\begin{array}{ll} {\ddot{\theta }}_{6} & =\frac{\cos\theta_{6}\;{\ddot{x}}_{\mathrm{tip}}+\sin\theta_{5}\sin\theta_{6}\;{\ddot{y}}_{\mathrm{tip}}-\cos\theta_{5}\sin\theta_{6}\;{\ddot{z}}_{\mathrm{tip}}}{l_{2}}-\frac{b_{6}}{ml_{2}^{2}}{\dot{\theta }}_{6}\\ & \;-\frac{l_{3}}{l_{2}^{2}}\sin\theta_{6}\;{\dot{\theta }}_{5}^{2}-\frac{g}{l_{2}}\cos\theta_{5}\sin\theta_{6} \end{array}$$ (2)

where $l_{3}=l_{1}+l_{2}\cos\left(\theta_{6}\right)$ , g denotes the gravitational acceleration and $m_{0}$ and m represent the masses of the two links of the suspended system, as illustrated in Figure 1. The parameters $b_{5}$ and $b_{6}$ denote the viscous damping coefficients associated with joints $\theta_{5}$ and $\theta_{6}$ , respectively. The quantities ${\ddot{x}}_{\mathrm{tip}}$ , ${\ddot{y}}_{\mathrm{tip}}$ , and ${\ddot{z}}_{\mathrm{tip}}$ represent the accelerations of the boom tip expressed in the base coordinate frame. These accelerations are obtained from the boom kinematics derived using Denavit–Hartenberg (DH) parameters Kalmari (2015); Kowsari and Ghabcheloo (2024).

The Cartesian position of the boom tip is given by Equation 3, and the radial and vertical coordinates are given by Equation 4.

$$p_{\mathrm{tip}}=\left(\begin{array}{c} r\cos\theta_{1}\\ r\sin\theta_{1}\\ z \end{array}\right)$$ (3)

where the radial distance r and vertical position z of the boom tip are obtained from the forward kinematics of the boom using the Denavit–Hartenberg parameters as:

$$\begin{array}{cc} & r=a_{2}-d_{4}\sin\left(\theta_{2}+\theta_{3}\right)+a_{4}\cos\left(\theta_{2}+\theta_{3}\right)+a_{3}\cos\left(\theta_{2}\right)\\ & z=d_{1}+d_{4}\cos\left(\theta_{2}+\theta_{3}\right)+a_{4}\sin\left(\theta_{2}+\theta_{3}\right)+a_{3}\sin\left(\theta_{2}\right) \end{array}$$ (4)

where $\theta_{2}$ and $\theta_{3}$ are the boom joint angles, $d_{1}$ and $d_{4}$ are DH displacement parameters, and $a_{2}$ , $a_{3}$ , and $a_{4}$ are DH geometric link parameters of the boom.

### 4.2 Cylindrical kinematic relations

The sway dynamics in (1)–(2) are written in terms of the boom-tip accelerations ${\ddot{x}}_{\mathrm{tip}}$ , ${\ddot{y}}_{\mathrm{tip}}$ , and ${\ddot{z}}_{\mathrm{tip}}$ . Since the practical motion commands of the boom are applied in cylindrical coordinates $\left(\theta_{1},r,z\right)$ , these accelerations must be expressed in terms of the corresponding cylindrical variables and their time derivatives. Differentiating the tip position (3) yields the Cartesian tip accelerations in Equations 5–7.

$${\ddot{x}}_{\mathrm{tip}}=\left(\ddot{r}-r{\dot{\theta }}_{1}^{2}\right)\cos\theta_{1}-\left(r{\ddot{\theta }}_{1}+2\dot{r}{\dot{\theta }}_{1}\right)\sin\theta_{1},$$ (5)

$${\ddot{y}}_{\mathrm{tip}}=\left(\ddot{r}-r{\dot{\theta }}_{1}^{2}\right)\sin\theta_{1}+\left(r{\ddot{\theta }}_{1}+2\dot{r}{\dot{\theta }}_{1}\right)\cos\theta_{1},$$ (6)

$${\ddot{z}}_{\mathrm{tip}}=\ddot{z}$$ (7)

substituting (7) into the sway dynamics shows that the angular accelerations ${\ddot{\theta }}_{5}$ and ${\ddot{\theta }}_{6}$ depend not only on the cylindrical velocity commands, but also on their second derivatives $\left({\ddot{\theta }}_{1},\ddot{r},\ddot{z}\right)$ . This dependence prevents the system from being directly written in the standard nonlinear state-space form required for predictive control.

This observation motivates the introduction of an input-consistent state transformation that removes the dependence on input accelerations while preserving the sway dynamics of the system.

### 4.3 Input-consistent state-space representation

The sway dynamics derived in Section 4.1 are expressed in terms of boom-tip accelerations. Under cylindrical task-space velocity commands $u={\left[{\dot{\theta }}_{1},\dot{r},\dot{z}\right]}^{T}$ , these accelerations contain the second derivatives $\left({\ddot{\theta }}_{1},\ddot{r},\ddot{z}\right)$ through the kinematic relations in (7).

If the system states are defined using the conventional generalized coordinates

$$x={\left[\theta_{5},{\dot{\theta }}_{5},\theta_{6},{\dot{\theta }}_{6},\theta_{1},r,z\right]}^{T}$$ (8)

the resulting dynamics explicitly depend on derivatives of the control inputs. Such a representation does not satisfy the standard nonlinear state-space form $\dot{x}=f\left(x,u\right)$ required for predictive control.

To eliminate the dependence on input accelerations and obtain a state-space model compatible with cylindrical velocity inputs, we introduce modified velocity states that embed the coupling between boom motion and sway dynamics.

### 4.4 State transformation

The transformed states are defined as

$$\begin{array}{cc} x_{1} & =\theta_{5},\\ x_{2} & ={\dot{\theta }}_{5}-\alpha_{5}\left(x\right){\dot{\theta }}_{1}-\alpha_{6}\left(x\right)\dot{r}-\alpha_{7}\left(x\right)\dot{z},\\ x_{3} & =\theta_{6},\\ x_{4} & ={\dot{\theta }}_{6}-\beta_{5}\left(x\right){\dot{\theta }}_{1}-\beta_{6}\left(x\right)\dot{r}-\beta_{7}\left(x\right)\dot{z},\\ x_{5} & =\theta_{1},\;x_{6}=r,\;x_{7}=z. \end{array}$$ (9)

The coefficients $\alpha_{i}\left(x\right)$ and $\beta_{i}\left(x\right)$ are not tuning parameters. They are obtained analytically by collecting the coefficients of the input accelerations ${\ddot{\theta }}_{1}$ , $\ddot{r}$ , and $\ddot{z}$ after substituting the cylindrical acceleration relations into the original sway dynamics. The role of the transformation is to absorb these input-acceleration terms into the modified velocity states. When the transformed states are differentiated, the input-acceleration terms appear with opposite signs and cancel exactly. As a result, the transformed model depends only on the state variables and the cylindrical velocity inputs. A detailed derivation and verification of this acceleration-cancellation procedure are provided in Supplementary Appendix A.

### 4.5 Resulting dynamics

Let the control input vector be defined as Equation 10

$$u={\left[\begin{array}{ccc} u_{1} & u_{2} & u_{3} \end{array}\right]}^{T}={\left[\begin{array}{ccc} {\dot{\theta }}_{1} & \dot{r} & \dot{z} \end{array}\right]}^{T}.$$ (10)

Differentiating the transformed states gives

$$\begin{array}{cc} {\dot{x}}_{1} & =x_{2}+\alpha_{5}\left(x\right)u_{1}+\alpha_{6}\left(x\right)u_{2}+\alpha_{7}\left(x\right)u_{3},\\ {\dot{x}}_{2} & =\varphi_{5}\left(x,u\right)-{\dot{\alpha }}_{5}\left(x\right)u_{1}-{\dot{\alpha }}_{6}\left(x\right)u_{2}-{\dot{\alpha }}_{7}\left(x\right)u_{3},\\ {\dot{x}}_{3} & =x_{4}+\beta_{5}\left(x\right)u_{1}+\beta_{6}\left(x\right)u_{2}+\beta_{7}\left(x\right)u_{3},\\ {\dot{x}}_{4} & =\varphi_{6}\left(x,u\right)-{\dot{\beta }}_{5}\left(x\right)u_{1}-{\dot{\beta }}_{6}\left(x\right)u_{2}-{\dot{\beta }}_{7}\left(x\right)u_{3},\\ {\dot{x}}_{5} & =u_{1},\;{\dot{x}}_{6}=u_{2},\;{\dot{x}}_{7}=u_{3}. \end{array}$$ (11)

The terms $\varphi_{5}\left(x,u\right)$ and $\varphi_{6}\left(x,u\right)$ denote the acceleration-free parts of the transformed sway dynamics. They are defined as

$$\varphi_{5}\left(x,u\right)≔{\ddot{\theta }}_{5}-\alpha_{5}\left(x\right){\ddot{\theta }}_{1}-\alpha_{6}\left(x\right)\ddot{r}-\alpha_{7}\left(x\right)\ddot{z},$$ (12)

$$\varphi_{6}\left(x,u\right)≔{\ddot{\theta }}_{6}-\beta_{5}\left(x\right){\ddot{\theta }}_{1}-\beta_{6}\left(x\right)\ddot{r}-\beta_{7}\left(x\right)\ddot{z}.$$ (13)

After substituting the expressions of $\alpha_{i}\left(x\right)$ and $\beta_{i}\left(x\right)$ , all occurrences of ${\ddot{\theta }}_{1}$ , $\ddot{r}$ , and $\ddot{z}$ cancel from $\varphi_{5}\left(x,u\right)$ and $\varphi_{6}\left(x,u\right)$ . Therefore, the final transformed model is driven only by the cylindrical velocity inputs $u={\left[{\dot{\theta }}_{1},\dot{r},\dot{z}\right]}^{T}$ , without requiring input accelerations. The system consequently admits the standard nonlinear state-space form

$$\dot{x}=f\left(x,u\right),$$ (14)

which is suitable for NMPC formulation.

### 4.6 Invertibility and validity domain

The proposed transformation is an input-dependent affine reparameterization of the sway velocity states. For a given admissible input

$$u={\left[\begin{array}{ccc} {\dot{\theta }}_{1} & \dot{r} & \dot{z} \end{array}\right]}^{T},$$

the original sway velocities can be recovered from the transformed states as

$${\dot{\theta }}_{5}=x_{2}+\alpha_{5}\left(x\right)u_{1}+\alpha_{6}\left(x\right)u_{2}+\alpha_{7}\left(x\right)u_{3},$$ (15)

$${\dot{\theta }}_{6}=x_{4}+\beta_{5}\left(x\right)u_{1}+\beta_{6}\left(x\right)u_{2}+\beta_{7}\left(x\right)u_{3}.$$ (16)

Thus, the transformation does not eliminate the sway velocity information; it only rewrites the velocity states in a form that cancels the input-acceleration terms.

Let the original state be written as

$$\xi ={\left[\begin{array}{ccccccc} \theta_{5} & \theta_{6} & \theta_{1} & r & z & {\dot{\theta }}_{5} & {\dot{\theta }}_{6} \end{array}\right]}^{T}.$$

For fixed u , the transformation from ξ to the transformed state is affine in ${\dot{\theta }}_{5}$ and ${\dot{\theta }}_{6}$ . Its Jacobian with respect to ξ has the block triangular form

$$\frac{\partial x}{\partial \xi }=\left[\begin{array}{cc} I_{5} & 0\\ ⋆ & I_{2} \end{array}\right],$$ (17)

where $I_{5}$ and $I_{2}$ are identity matrices and ⋆ contains derivatives of the coefficient functions. Therefore,

$$\det\left(\frac{\partial x}{\partial \xi }\right)=1,$$ (18)

which shows that the transformation is locally invertible wherever the coefficient functions are finite and smooth.

The possible singularities are associated with the underlying physical and coordinate definitions, not with the affine velocity reparameterization itself. The coefficient functions contain the denominators

$$I_{5}=m_{0}l_{0}^{2}+ml_{3}^{2},\;l_{2},\;ml_{2}^{2},$$ (19)

where

$$l_{3}=l_{1}+l_{2}\cos\theta_{6}.$$ (20)

Hence, the transformed model is well-posed for non-degenerate suspended-load parameters satisfying

$$m>0,\;l_{2}>0,\;I_{5}>0.$$

Moreover, because the boom-tip position is expressed in cylindrical coordinates, the formulation assumes operation away from the cylindrical-coordinate singularity r=0 . This condition is satisfied in the considered hydraulic-boom operating range, where the boom operates with positive radial extension and within its mechanical limits. Therefore, the proposed transformed model is well-posed over the admissible operating domain considered in this study.

### 4.7 State availability and observability

The transformed velocity states $x_{2}$ and $x_{4}$ are not directly measurable physical quantities. They are algebraic reparameterizations of the physical sway velocities. For a given known input

$$u={\left[\begin{array}{ccc} {\dot{\theta }}_{1} & \dot{r} & \dot{z} \end{array}\right]}^{T},$$

the transformed states are computed from the physical sway states as

$$x_{2}={\dot{\theta }}_{5}-\alpha_{5}\left(x\right)u_{1}-\alpha_{6}\left(x\right)u_{2}-\alpha_{7}\left(x\right)u_{3},$$ (21)

$$x_{4}={\dot{\theta }}_{6}-\beta_{5}\left(x\right)u_{1}-\beta_{6}\left(x\right)u_{2}-\beta_{7}\left(x\right)u_{3}.$$ (22)

Therefore, the transformed states do not require direct measurement. They can be reconstructed algebraically once the physical sway states, boom-tip coordinates, and command inputs are available.

For practical implementation, the required physical state vector can be written as

$$\xi ={\left[\begin{array}{ccccccc} \theta_{5} & {\dot{\theta }}_{5} & \theta_{6} & {\dot{\theta }}_{6} & \theta_{1} & r & z \end{array}\right]}^{T}.$$

If an estimate $\hat{\xi }$ is available from sensors or from a state observer, the corresponding transformed state estimate is obtained as

$$\hat{x}=T\left(\hat{\xi },u\right).$$ (23)

Since the transformation is locally invertible over the admissible operating domain, it does not reduce the information content of the original physical state. Consequently, observability of the transformed representation is equivalent to observability of the original physical sway model under the same measurement configuration.

In the simulations presented in this work, the full state is available from the simulation model. In a real hydraulic boom implementation, the sway states must be supplied either by direct sensing, for example, using inertial or vision-based payload-sway measurements, or by a nonlinear state observer such as an extended Kalman filter or moving-horizon estimator. The design and experimental validation of such an observer are outside the scope of the present work and are identified as future work.

## 5 Proposed nonlinear model predictive control of the boom–grapple system

This section presents NMPC framework developed for the hydraulic boom equipped with an underactuated grapple. The controller aims to simultaneously achieve accurate goal reaching of the boom tip, suppression of grapple sway, and safe motion through obstacle avoidance.

### 5.1 NMPC formulation

NMPC is adopted because it allows optimization of control inputs under nonlinear system dynamics and operational constraints over a finite prediction horizon Mayne (2014); Rawlings et al. (2020). At each sampling instant, the controller predicts the system evolution and computes a sequence of control inputs that minimizes a predefined cost function while satisfying the system constraints.

In hydraulic boom systems with suspended loads, a fundamental trade-off exists between rapid boom motion and suppression of sway oscillations. Fast boom movements excite the pendulum dynamics of the grapple, whereas aggressive sway damping may slow down task execution. The NMPC cost function is therefore designed to balance these competing objectives by penalizing motion increments, control effort, sway motion, and terminal goal error. It should be noted that the proposed NMPC is formulated as a point-to-point goal-reaching controller rather than only as a reference-trajectory-following controller. The controller is initialized from the current boom state and computes a feasible motion toward the desired goal position while accounting for actuator limits, obstacle avoidance, input smoothness, and grapple-sway suppression. This formulation affects the cost-function design: instead of only penalizing deviation from a predefined time-indexed reference trajectory, the objective balances goal reaching, motion efficiency, terminal positioning accuracy, and residual sway near the final picking configuration.

The resulting NMPC optimization problem is formulated as

$$\begin{array}{cc} \underset{u_{0:N-1}}{\min}\; & \frac{1}{N}\overset{N-1}{\underset{k=0}{\sum }}\left(\|\Delta p_{k}{\|}_{Q}^{2}+\|u_{k}{\|}_{R_{u}}^{2}+\|\Delta u_{k}{\|}_{R}^{2}\right.\\ & \left.\;+\|e_{\theta ,k}{\|}_{Q_{\theta }}^{2}+\|e_{\dot{\theta },k}{\|}_{Q_{\dot{\theta }}}^{2}\right)+J_{f}\\ \text{s.t.}\; & x_{k+1}=f\left(x_{k},u_{k}\right)\\ & u_{\min}\leq u_{k}\leq u_{\max}\\ & d\left(x_{k},x_{\mathrm{obs}}\right)\geq d_{\min} \end{array}$$ (24)

where N denotes the prediction horizon, $x_{k}$ is the system state at prediction step k , $u_{k}$ is the cylindrical velocity input vector, $\Delta p_{k}$ is the incremental boom-tip motion, $\Delta u_{k}$ is the input increment, $e_{\theta ,k}$ and $e_{\dot{\theta },k}$ are the sway-angle and sway-rate error vectors, respectively, and $J_{f}$ is the terminal cost. The matrices Q , $R_{u}$ , R , $Q_{\theta }$ , and $Q_{\dot{\theta }}$ are positive semi-definite weighting matrices used to balance boom motion, control effort, input smoothness, sway suppression, and sway-rate damping. The bounds $u_{\min}$ and $u_{\max}$ define the actuator velocity limits, while $d\left(x_{k},x_{\text{obs}}\right)$ denotes the distance between the grapple and the obstacle, and $d_{\min}$ is the minimum allowed safety distance.

The NMPC prediction model uses the input-consistent state-space representation derived in Section 4.3. The state vector $x_{k}$ corresponds to the transformed states defined in Equation 11, which capture the sway dynamics of the grapple together with the cylindrical coordinates of the boom tip.

The control input vector is defined as

$$u_{k}={\left[{\dot{\theta }}_{1},\dot{r},\dot{z}\right]}^{T}$$ (25)

which corresponds to the cylindrical task-space velocity commands of the boom.

### 5.2 Cost function components

The incremental motion term

$$\Delta p_{k}=p_{k}-p_{k-1}$$ (26)

where $p_{k}={\left[\theta_{1,k},r_{k},z_{k}\right]}^{T}$ denotes the cylindrical task-space coordinates of the boom tip, penalizes rapid changes in boom motion. This encourages smooth trajectory evolution and reduces excitation of the suspended load.

The control effort term $\|u_{k}{\|}_{R_{u}}^{2}$ limits excessive actuator inputs, while the input increment term

$$\Delta u_{k}=u_{k}-u_{k-1}$$ (27)

penalizes abrupt control changes and promotes smoother actuator behavior.

The sway error terms $e_{\theta ,k}$ and $e_{\dot{\theta },k}$ represent the angular displacement and angular velocity of the underactuated sway joints. Penalizing these quantities suppresses oscillatory motion of the suspended grapple. The sway error vectors are defined as:

$$e_{\theta ,k}=\left[\begin{array}{c} \theta_{5,k}\\ \theta_{6,k} \end{array}\right],\;e_{\dot{\theta },k}=\left[\begin{array}{c} {\dot{\theta }}_{5,k}\\ {\dot{\theta }}_{6,k} \end{array}\right].$$ (28)

### 5.3 Terminal cost and rationale

To regulate the trade-off between goal reaching and sway suppression, a state-dependent terminal cost is introduced as

$$J_{f}=\left(1-\tanh\left(a\|e_{x,N}{\|}^{2}\right)\right)\|e_{\theta ,N}{\|}_{Q_{f}^{\theta }}^{2}+\|e_{x,N}{\|}_{Q_{x}}^{2},$$ (29)

where $e_{x,N}$ denotes the boom-tip position error relative to the target at the final prediction step, $e_{\theta ,N}$ represents the terminal sway-angle error, and a>0 controls the activation rate of the terminal sway penalty.

The terminal cost is motivated by the point-to-point nature of the forwarder log-picking task. During the main maneuver, productivity requires sufficiently efficient boom motion toward the log-picking location. Enforcing near-zero sway throughout the entire motion would make the controller overly conservative and could significantly slow down the maneuver. Therefore, sway suppression should not dominate the objective when the boom is still far from the target. However, near the final picking configuration, low residual sway becomes critical because the grapple must approach the log with small oscillations to enable accurate and reliable grasping.

The scalar function

$$w\left(e_{x,N}\right)=1-\tanh\left(a\|e_{x,N}{\|}^{2}\right)$$ (30)

acts as a smooth distance-dependent activation of the terminal sway penalty. Since

$$0\leq w\left(e_{x,N}\right)\leq 1,$$ (31)

the sway penalty is smoothly reduced when the boom is far from the target and progressively increased as the boom approaches the target. When $\left\|e_{x,N}\right\|$ is large, $w\left(e_{x,N}\right)$ is close to zero, allowing the optimizer to prioritize motion toward the log-picking location. When $\left\|e_{x,N}\right\|$ becomes small, $w\left(e_{x,N}\right)$ approaches one, and the terminal sway penalty becomes fully active.

The terminal position penalty $\|e_{x,N}{\|}_{Q_{x}}^{2}$ remains active throughout the maneuver and drives the boom tip toward the target. The terminal sway penalty is activated near the goal, where residual oscillations are most relevant for accurate positioning and log picking. Thus, the terminal cost provides a smooth state-dependent scalarization of two competing objectives: motion efficiency and terminal sway suppression.

It should be noted that this terminal cost is an application-motivated performance term and should not be interpreted as a Lyapunov terminal penalty. A formal stability guarantee would require additional terminal ingredients, such as a terminal invariant set and a locally stabilizing terminal controller. These extensions are left for future work.

The weighting matrices Q , R , $R_{u}$ , $Q_{\theta }$ , $Q_{\dot{\theta }}$ , $Q_{f}^{\theta }$ , and $Q_{x}$ are selected to balance goal accuracy, sway suppression, actuator effort, input smoothness, and terminal positioning accuracy.

The controller parameters were selected as an application-oriented tuning to demonstrate the proposed reduced-order NMPC framework. They should not be interpreted as globally optimal weights. The main role of the weights is to regulate the trade-off among goal reaching, sway suppression, actuator effort, and input smoothness. Increasing the sway-related weights generally improves sway damping but leads to more conservative and slower boom motion. Conversely, reducing these weights allows faster motion but may increase residual oscillations near the target. Similarly, larger input and input-increment penalties produce smoother velocity commands but may reduce motion aggressiveness.

A complete robustness and sensitivity analysis with respect to all tuning parameters, model parameters, actuator dynamics, payload properties, obstacle configurations, and initial sway conditions is outside the scope of the present work. The focus of this study is the reduced-order input-consistent state-space reformulation and its use as an NMPC prediction model. Systematic robustness analysis and automatic tuning of the NMPC weights are left for future work.

### 5.4 Constraints

Actuator limits: The cylindrical velocity inputs are bounded by the physical limits of the hydraulic actuators:

$$u_{\min}\leq u_{k}\leq u_{\max}$$ (32)

Obstacle avoidance: Obstacle avoidance is enforced by maintaining a minimum safe distance between the grapple and obstacles in the workspace:

$$d\left(x_{k},x_{\mathrm{obs}}\right)\geq d_{\min}$$ (33)

here $d\left(x_{k},x_{\mathrm{obs}}\right)$ denotes the Euclidean distance between the grapple position and the obstacle location, and $d_{\min}$ represents a predefined safety margin ensuring collision-free boom motion.

The proposed NMPC formulation differs from conventional reference-tracking approaches in two main aspects. First, it uses the input-consistent state-space representation developed in Section 4.3, which allows direct use of cylindrical velocity commands while capturing the coupled sway dynamics. Second, the controller is formulated as an integrated point-to-point motion planner and controller. It generates a feasible motion from the current boom configuration to the log-picking target while accounting for obstacle avoidance, actuator limits, input smoothness, and sway suppression.

## 6 Actuator dynamics

In practical hydraulic manipulators, the commanded velocity inputs cannot be applied instantaneously due to the dynamics of the hydraulic actuators. These dynamics introduce delays and limited response rates that affect the overall behavior of the boom–grapple system.

To capture this effect, the actuator dynamics are incorporated into the system model using a first-order representation. In real hydraulic manipulators, actuators exhibit delays and limited response rates due to hydraulic and mechanical constraints. Ignoring these dynamics may lead to control commands that are infeasible for the physical system.

The actuator dynamics are modeled as first-order systems in Equation 34:

$$\begin{array}{cc} {\dot{x}}_{act_{1}} & =-\frac{1}{\tau_{1}}x_{act_{1}}+k_{1}u_{1}\\ {\dot{x}}_{act_{2}} & =-\frac{1}{\tau_{2}}x_{act_{2}}+k_{2}u_{2}\\ {\dot{x}}_{act_{3}} & =-\frac{1}{\tau_{3}}x_{act_{3}}+k_{3}u_{3} \end{array}$$ (34)

where $x_{act_{i}}$ represents the actuator output corresponding to control input $u_{i}$ , and $k_{i}$ and $\tau_{i}$ denote the actuator gain and time constant, respectively.

The actuator parameters $k_{i}$ and $\tau_{i}$ were identified from a high-fidelity AMESim model of an industrial forwarder crane. The AMESim model was used as an engineering simulator to characterize the dominant hydraulic actuator response of the boom system. Due to industrial confidentiality, the manufacturer, exact machine model, detailed AMESim schematic, and proprietary hydraulic specifications are not disclosed in this manuscript. Nevertheless, the simulator represents the hydraulic actuator response and boom-motion behavior of a commercial forwarder-crane platform and provides a realistic engineering environment for evaluating the proposed reduced-order prediction model before real-machine implementation.

**FIGURE 2:**
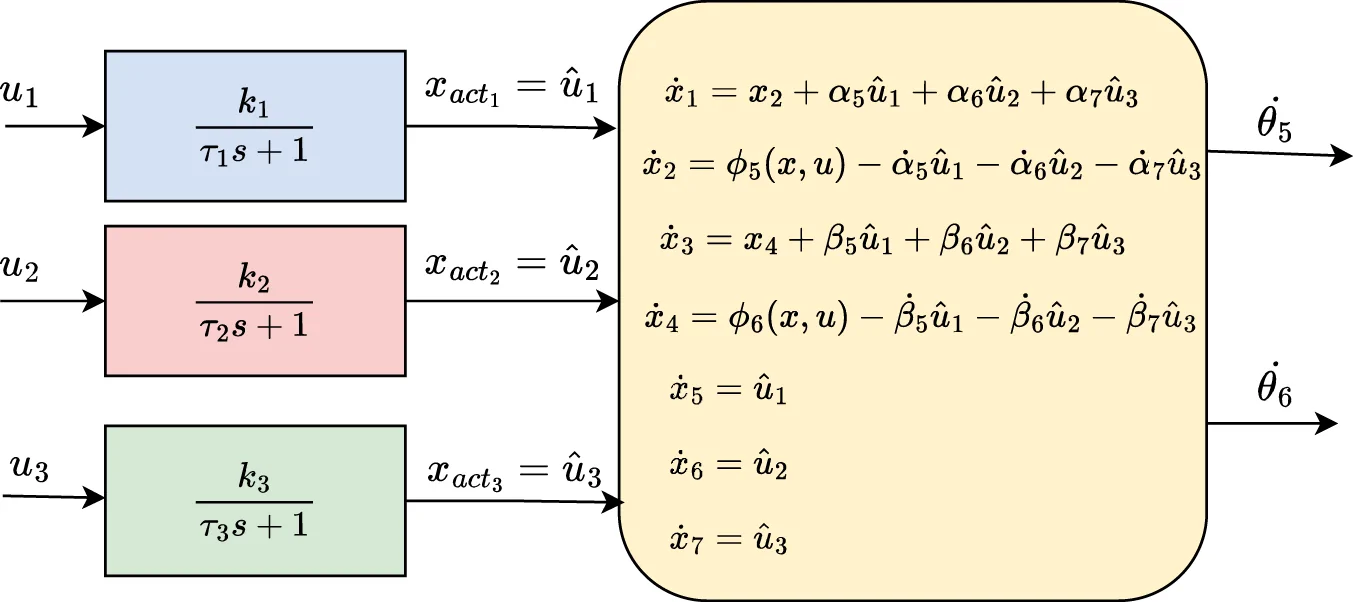
Control architecture incorporating actuator dynamics. The commanded cylindrical velocity inputs are processed through first-order actuator models before being applied to the boom–grapple state-space system.

By incorporating these dynamics into the prediction model, the controller accounts for actuator response delays, leading to more realistic control commands and improved stability of the boom motion. Figure 2 illustrates the resulting control structure, where the input commands are processed through the actuator models before being applied to the state-space system.

**FIGURE 3:**
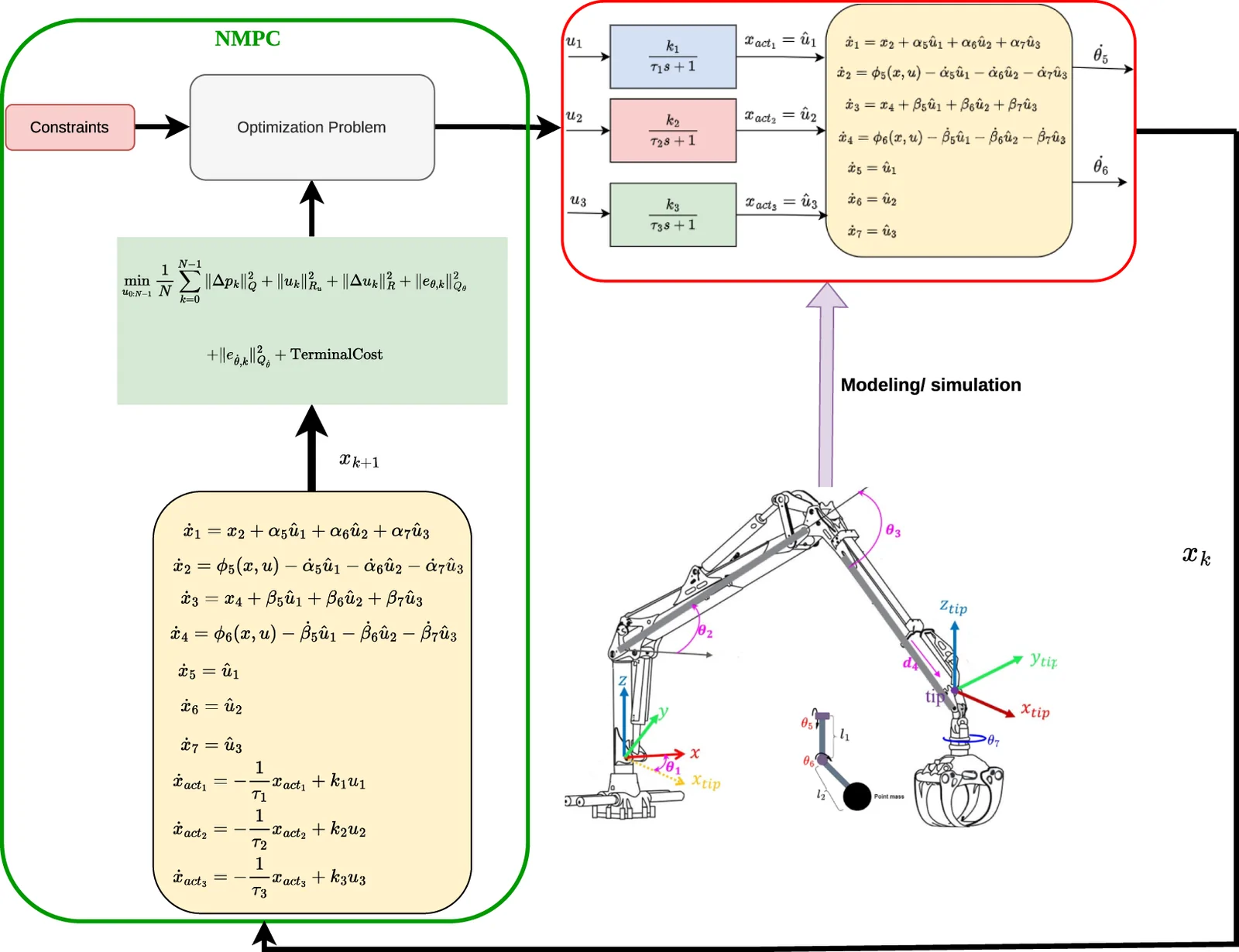
Control architecture of the proposed NMPC framework.

The overall control architecture of the proposed NMPC framework, including the actuator dynamics and the boom–grapple state-space model, is illustrated in Figure 3.

## 7 Computational and modeling considerations

### 7.1 Modeling assumptions and validity

The NMPC prediction model used in this work is control-oriented. The suspended grapple is modeled as a two-degree-of-freedom pendulum attached to the boom tip. This structure is physically consistent with the considered grapple suspension, where the dominant passive sway motions are represented by the angles $\theta_{5}$ and $\theta_{6}$ . The model is derived from the equations of motion and includes the relevant inertial terms, gravitational restoring effects, viscous damping at the passive joints, and excitation due to boom-tip accelerations.

The main modeling assumption is related to the representation of the suspended payload configuration. In the present formulation, the grapple/load system is represented using lumped inertial parameters, and the effective center of mass is assumed to be located at the grapple-side suspended body. Variations caused by different log masses, log geometries, log orientations, or shifts in the combined grapple-log center of mass are not explicitly modeled. This assumption is suitable for deriving and evaluating the proposed reduced-order input-consistent formulation, but it may affect the quantitative accuracy of the predicted sway response under large payload variations.

The actuator dynamics are represented using first-order models. This approximation captures the dominant response delay and limited bandwidth of the hydraulic actuators, which is important because commanded cylindrical velocities cannot be applied instantaneously. The parameters of these first-order models were identified from a high-fidelity AMESim model of an industrial forwarder crane. Therefore, the actuator model provides a practical control-oriented approximation of the actuator response while keeping the NMPC prediction problem computationally manageable.

These assumptions mainly affect the quantitative accuracy of the predicted response under operating conditions involving large payload variation, changing log center-of-mass location, strong hydraulic nonlinearities, external impacts, structural flexibility, frictional effects, or operation outside the identified actuator range. They do not affect the main theoretical contribution of the paper, namely, the reduced-order input-consistent state-space reformulation and the analytical cancellation of input accelerations. Future work will extend the validation to payload-dependent inertial parameters, varying log center-of-mass locations, higher-fidelity actuator models, and experimental data from hydraulic boom operation.

### 7.2 Analytical computational complexity

The computational implication of the proposed reduced-order model was evaluated analytically by comparing the size of the resulting NMPC optimization problem. For a multiple-shooting NMPC formulation, the number of decision variables scales with the state and input dimensions as according to Equation 35

$$n_{\text{var}}=\left(N+1\right)n_{x}+Nn_{u},$$ (35)

where $n_{x}$ is the number of states, $n_{u}$ is the number of control inputs, and N is the prediction horizon.

Using the prediction horizon N=80 , the proposed 7-state model with three cylindrical velocity inputs gives 807 decision variables. In contrast, representative 10-state and 12-state formulations lead to 1050 and 1292 decision variables, respectively. The corresponding number of dynamic equality constraints is reduced from 800 to 960 to 560. This reduction directly decreases the size of the nonlinear program solved at each NMPC step.

The actuator dynamics included in the proposed framework are implementation-related and should not be interpreted as a disadvantage of the reduced-order reformulation. If comparable first-order actuator-response dynamics are included consistently for all formulations, one actuator state is added per independent input channel. The proposed formulation then has 10 states, the Cartesian-tip formulation has 13 states, and the joint-space formulation has 16 states. With N=80 , the corresponding decision-variable counts are 1050, 1293, and 1616, respectively. Therefore, the reduction in NMPC problem size is preserved even when actuator dynamics are considered for practical implementation. The corresponding NMPC problem-size comparison is summarized in Table 2.

**TABLE 2:** Analytical comparison of NMPC problem size for the proposed and representative alternative formulations with N=80 .

Formulation	Core states	Inputs	States with actuator dynamics	Decision variables, core	Decision variables, actuator-augmented
Proposed cylindrical	7	3	10	807	1050
Cartesian-tip	10	3	13	1050	1293
Joint-space	12	4	16	1292	1616

## 8 Simulation results

To evaluate the effectiveness of the proposed NMPC framework and the input-consistent state-space representation, a series of simulation studies were conducted. The simulations assess the controller’s ability to achieve accurate goal reaching, suppress sway motion of the suspended grapple, and avoid obstacles during boom operation.

### 8.1 Simulation setup and scenarios

The sampling time in all simulations was set to 0.02 s. Controller parameters were tuned empirically to achieve a balance between tracking accuracy, sway suppression, and control smoothness. After empirical tuning, a prediction horizon of N=80 was selected as a compromise between computational efficiency and control performance. The weighting matrices used in the NMPC cost function were selected as $Q=10I_{3}$ , $R=100I_{3}$ , $R_{u}=50I_{3}$ , $Q_{\theta }=50I_{2}$ , and $Q_{x}=500I_{3}$ , where $I_{n}$ denotes an identity matrix of dimension n .

To evaluate different aspects of controller performance, two representative simulation scenarios were designed. The first scenario evaluates obstacle avoidance capability, while the second examines the influence of actuator dynamics on control performance.

### 8.2 Scenario 1: obstacle avoidance

To represent obstacles commonly encountered in forestry operations, two obstacle types were considered: a cylindrical object representing a tree trunk and a planar obstacle representing a wall or tray.

The cylindrical obstacle is approximated using a set of equal-sized spheres (five in this study), which enables efficient distance evaluation within the NMPC optimization problem. The plate obstacle introduces constraints along the vertical direction, representing environmental boundaries in the workspace.

Since the available measurements include only the boom-tip position, velocity, and acceleration, the grapple position is estimated using its kinematic relationship with the boom tip. By deriving the transformation from the boom tip to the grapple center of mass, the system can estimate the grapple position without additional sensors. The position of the grapple in the world frame is given by Equation 36

$$\begin{array}{cc} x_{tm} & =x_{\text{tip}}-l_{2}\sin\theta_{6}\cos\theta_{1}-l_{3}\sin\theta_{6}\sin\theta_{1},\\ y_{tm} & =y_{\text{tip}}-l_{2}\sin\theta_{6}\sin\theta_{1}+l_{3}\sin\theta_{6}\cos\theta_{1},\\ z_{tm} & =z_{\text{tip}}-l_{3}\cos\theta_{6} \end{array}$$ (36)

Collision avoidance is enforced by maintaining a minimum distance between the spherical representation of the grapple and surrounding obstacles defined in Equation 37

$$\sqrt{{\left(x_{tm}-x_{\text{obs},i}\right)}^{2}+{\left(y_{tm}-y_{\text{obs},i}\right)}^{2}+{\left(z_{tm}-z_{\text{obs},i}\right)}^{2}}\geq \left(r_{\text{obs},i}+r_{m}\right),\;i=1,2,3.$$ (37)

**FIGURE 4:**
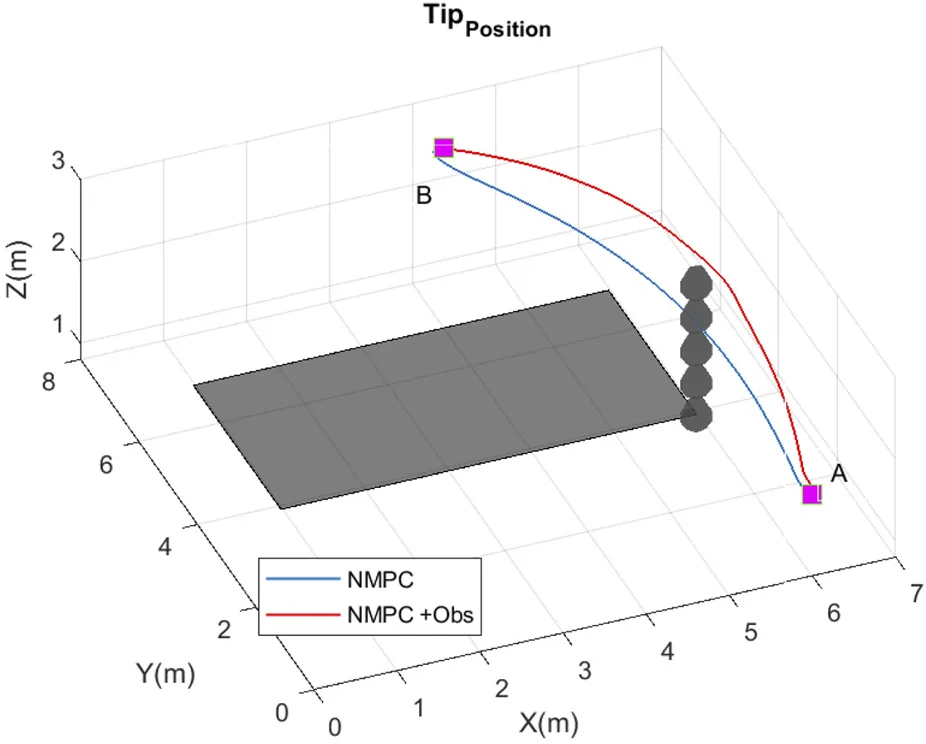
3D trajectory of the boom tip with and without obstacle avoidance. The nominal NMPC path (blue) reaches the goal directly, while the presence of an obstacle (black sphere) causes the controller to generate an alternative collision-free trajectory (red).

**FIGURE 5:**
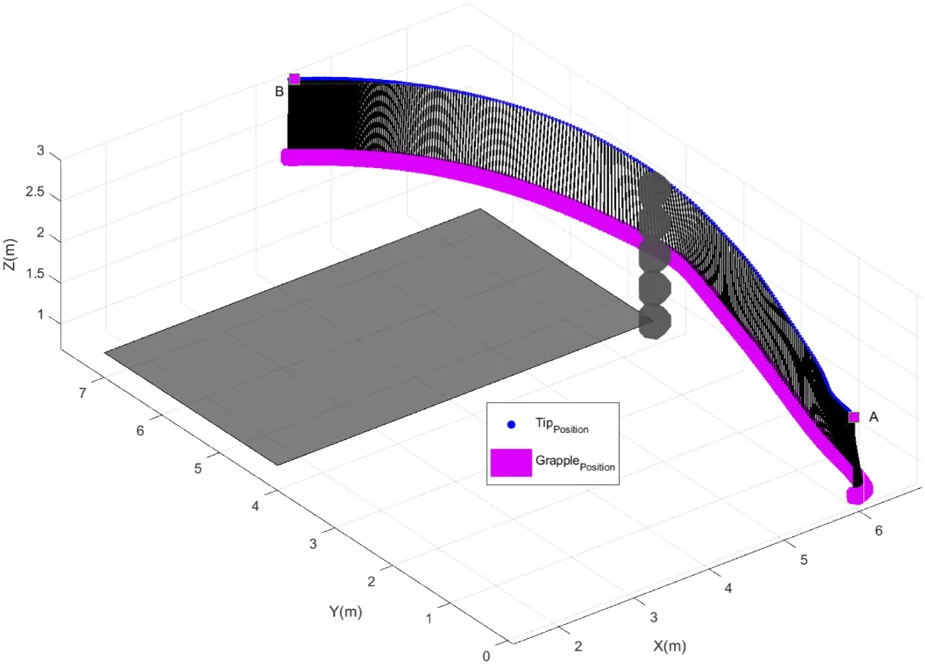
3D trajectory of the boom tip using the proposed NMPC controller.

**FIGURE 6:**
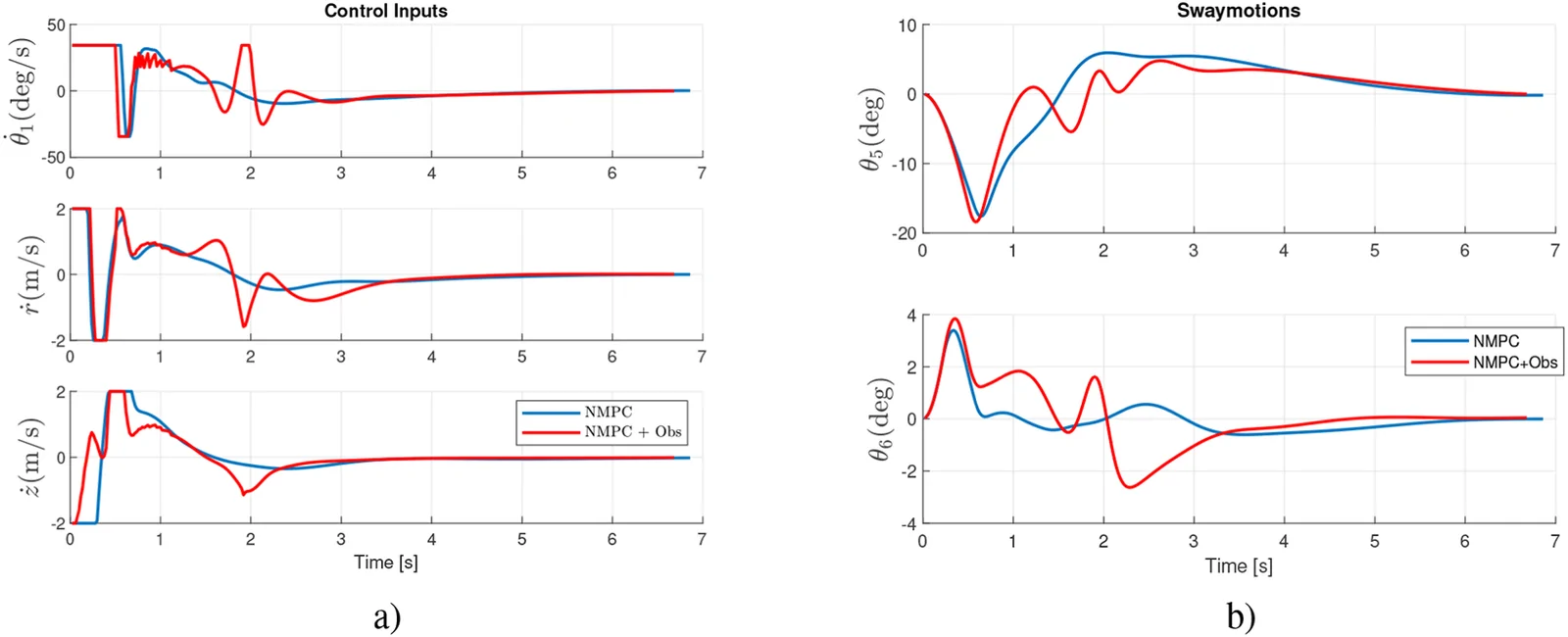
Comparison of control inputs and sway motion using NMPC with and without obstacle constraints. **(a)** Control inputs $\left({\dot{\theta }}_{1},\dot{r},\dot{z}\right)$ . **(b)** Sway motion of the underactuated joints $\left(\theta_{5},\theta_{6}\right)$ .

To demonstrate obstacle avoidance capability, the NMPC controller was first simulated without obstacles to obtain a reference trajectory between the start and goal positions. An obstacle was then placed directly on this trajectory, and the NMPC optimization was repeated. The resulting boom-tip trajectories with and without obstacle avoidance are shown in Figure 4, and the corresponding boom-tip and grapple trajectories are shown in Figure 5. Figure 6 compares the corresponding control inputs and sway responses obtained using the proposed NMPC controller with and without obstacle constraints.

### 8.3 Scenario 2: sensitivity to actuator dynamics

In practical hydraulic manipulators, actuator dynamics influence the response speed of the boom and therefore affect sway behavior and tracking performance. To evaluate this effect, a sensitivity analysis was performed by varying actuator time constants.

Actuator dynamics were identified using a high-fidelity AMESim model of an industrial forwarder crane. Due to industrial confidentiality, the manufacturer and exact machine model are not disclosed. Nevertheless, the simulator represents the hydraulic actuator response and boom-motion behavior of a commercial forwarder-machine platform and provides a realistic engineering environment for evaluating the proposed reduced-order prediction model before real-machine implementation.

The identified actuator models were characterized by their time constants τ and gain parameters. These models were incorporated into the NMPC prediction model to reflect realistic actuator behavior.

**FIGURE 7:**
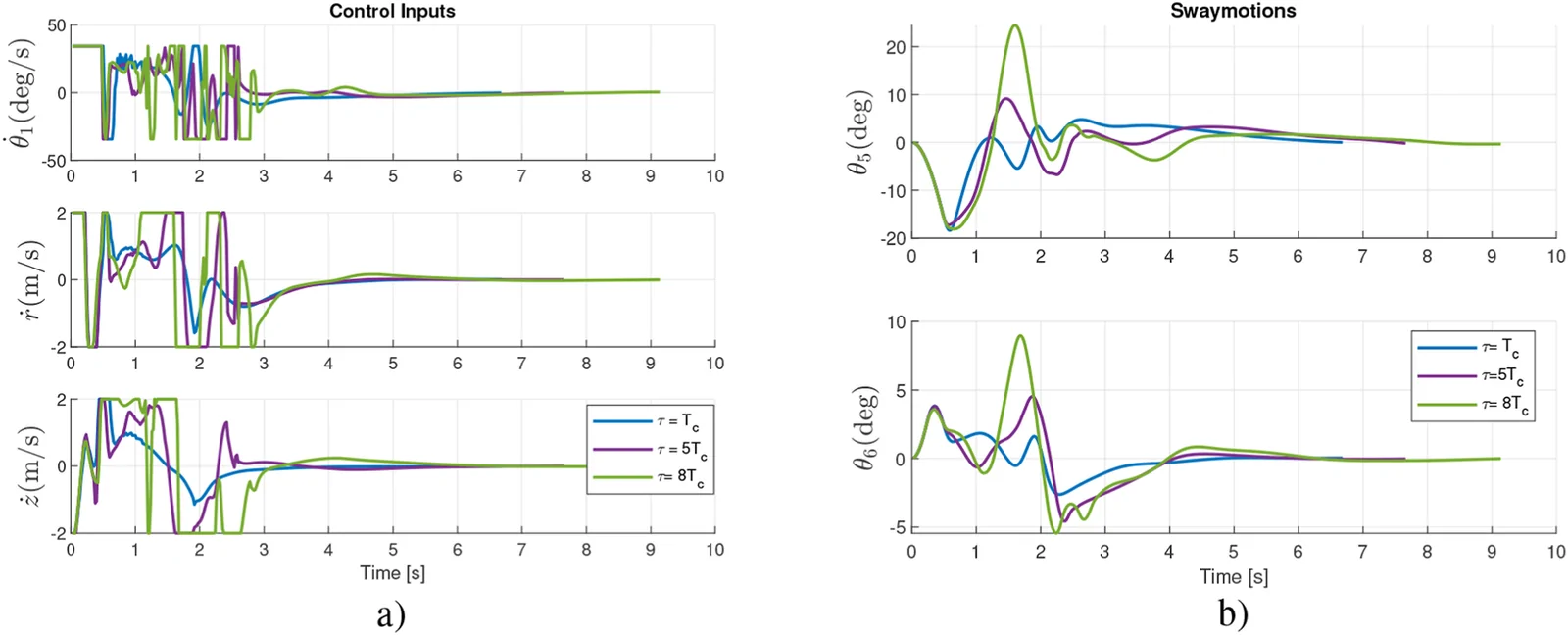
Control inputs and sway motions for varying actuator time constants. **(a)** Control inputs ( ${\dot{\theta }}_{1},\;\dot{r}\dot{z}$ ). **(b)** Sway motions of the underactuated joints (θ_5_,θ_6_). Larger actuator time constants result in slower response, increased sway oscillations, and delayed convergence to the target.

Controller performance was evaluated using the following metrics:

- Goal-reaching accuracy: final position error at the target
- Residual sway: amplitude of oscillations after reaching the goal
- Control effort: magnitude and smoothness of control inputs. The effect of varying actuator time constants on the control inputs and sway motions is shown in Figure 7

The results show that increasing actuator time constants significantly affects system performance. Larger values of τ lead to slower actuator responses, which results in more oscillatory control inputs, increased sway amplitudes, and delayed convergence to the desired goal position.

These observations highlight the importance of incorporating actuator dynamics into the predictive control model. Ignoring actuator dynamics can lead to unrealistic control commands and degraded system performance. The computed performance metrics for the different actuator time constants are reported in Table 3.

**TABLE 3:** Performance metrics under different actuator time constants.

τ	Max sway	Tracking error	Stability	Time to reach accuracy ( <0.01 m)
$\tau =T_{c}$	Moderate	Low	Stable	<7s
$\tau =5T_{c}$	High	Medium	Marginal	7.6s
$\tau =8T_{c}$	Very high	High	Poor	>9s

### 8.4 Performance metrics

To provide an objective evaluation of the NMPC performance, quantitative metrics were computed for the simulation scenarios. Since the considered task is a point-to-point boom motion from the current configuration to a desired log-picking location, the final positioning accuracy was evaluated as Equation 38

$$e_{p,f}=\|p\left(t_{f}\right)-p_{\text{goal}}\|,$$ (38)

where $p\left(t_{f}\right)$ is the final boom-tip position and $p_{\text{goal}}$ is the target position. The sway behavior was evaluated using the maximum sway amplitude and the RMS sway angle,

$$\theta_{\text{max}}=\underset{t\in \left[0,T\right]}{\max}\sqrt{\theta_{5}^{2}\left(t\right)+\theta_{6}^{2}\left(t\right)},$$ (39)

$$\theta_{\text{RMS}}=\sqrt{\frac{1}{T}\int_{0}^{T}\left(\theta_{5}^{2}\left(t\right)+\theta_{6}^{2}\left(t\right)\right)dt}.$$ (40)

In addition, the residual sway near the goal was evaluated over a terminal window [T−ΔT,T] , since low residual sway near the final picking configuration is critical for accurate log grasping:

$$\theta_{\text{res}}=\sqrt{\frac{1}{\Delta T}\int_{T-\Delta T}^{T}\left(\theta_{5}^{2}\left(t\right)+\theta_{6}^{2}\left(t\right)\right)dt}.$$ (41)

The control effort and input smoothness were quantified as

$$J_{u}=\int_{0}^{T}u^{T}\left(t\right)u\left(t\right)\;dt,$$ (42)

$$J_{\Delta u}=\int_{0}^{T}{\dot{u}}^{T}\left(t\right)\dot{u}\left(t\right)\;dt.$$ (43)

For the obstacle-avoidance scenario, the minimum obstacle clearance was also computed as

$$d_{\min}^{\text{obs}}=\underset{t\in \left[0,T\right]}{\min}d\left(x\left(t\right),x_{\text{obs}}\right).$$ (44)

These metrics provide quantitative evaluation of goal-reaching accuracy, sway suppression, control effort, input smoothness, maneuver duration, and obstacle-constraint satisfaction. The closed-loop simulation was terminated when the boom reached the prescribed goal region, defined by a cylindrical-coordinate tolerance of 0.05 m , or when the maximum simulation time was reached. The final Cartesian boom-tip error is reported explicitly in Table 4; therefore, the reported maneuver time corresponds to reaching this practical goal region rather than exact asymptotic convergence.

**TABLE 4:** Quantitative performance evaluation of the proposed NMPC controller.

Scenario	$e_{p,f}$ [m]	$\theta_{\max}$ [deg]	$\theta_{\text{RMS}}$ [deg]	$\theta_{\text{res}}$ [deg]	$J_{u}$	$J_{\Delta u}$	$t_{\text{man}}$ [s]
NMPC without obstacle	0.071	26.20	7.78	1.03	3.013	75.361	5.46
NMPC with obstacle	0.054	22.35	6.60	1.95	4.748	155.722	5.04

The reported maneuver time denotes the physical closed-loop task duration and should not be interpreted as NMPC solver execution time. The results show that the proposed NMPC reaches the desired goal with final boom-tip errors below 0.08 m in both scenarios. The residual sway near the final configuration remains below 2° , indicating that the controller reduces the oscillation level near the log-picking position. In the obstacle-avoidance scenario, the control effort and input-smoothness cost increase, which is expected because the optimizer must satisfy the additional obstacle-avoidance constraints. The computed minimum obstacle clearance is approximately zero within numerical precision, indicating that the obstacle constraint becomes active without meaningful penetration.

## 9 Conclusion

This paper presented a NMPC framework for the control of hydraulic booms equipped with an underactuated grapple. The main contribution of the work is a reduced-order state-space reformulation of the grapple sway dynamics expressed directly in cylindrical task-space velocity coordinates, which correspond to the practical command interface of hydraulic boom systems.

The proposed input-consistent state transformation eliminates the dependence of the dynamics on input accelerations and yields a nonlinear model driven directly by cylindrical velocity commands. Based on this representation, an NMPC controller was developed to simultaneously achieve accurate goal reaching, sway suppression, and obstacle avoidance. The NMPC cost function was designed to capture the practical trade-off between rapid boom motion and suppression of suspended load oscillations, enabling efficient motion while progressively damping sway near the target configuration. In addition, first-order actuator dynamics identified from an AMESim model of a forwarder crane were incorporated to better reflect realistic actuator behavior.

Simulation results demonstrated that the proposed approach effectively reduces sway motion of the suspended grapple while maintaining accurate task-space motion of the boom. The controller was able to generate collision-free trajectories in the presence of obstacles and maintain stable performance under varying actuator dynamics. These results indicate that the proposed cylindrical state-space reformulation simplifies predictive control design for hydraulic booms while preserving the essential sway dynamics of suspended loads.

Future work will focus on experimental validation of the proposed framework on a real hydraulic manipulator platform and on extending the approach to more complex environments involving multiple obstacles and additional operational constraints.

A systematic closed-loop comparison with joint-space MPC, Cartesian-tip NMPC, and other anti-sway controllers under identical implementation assumptions remains an important direction for future work.

## Data Availability

The original contributions presented in the study are included in the article/Supplementary Material, further inquiries can be directed to the corresponding author.
