# Supplementary material for: Reduced-order state-space reformulation for task-space predictive control of an under-actuated hydraulic boom
Source: Front Robot AI. 2026 Aug 6;13:1853243. doi: 10.3389/frobt.2026.1853243 (PMC13527712; doi:10.3389/frobt.2026.1853243)
Supplement: Supplementary file 1 [file Supplementaryfile1.pdf]

Supplementary Appendix A

Derivation and verification of the input-consistent state transformation

Elham Kowsari and Reza Ghabcheloo

Appendix A: Derivation and verification of the input-consistent transformation

This appendix provides the derivation of the input-consistent state transformation used in the main manuscript. The control inputs are the cylindrical velocity commands

$$u = \begin{bmatrix} u_1 & u_2 & u_3 \end{bmatrix}^T = \begin{bmatrix} \dot{\theta}_1 & \dot{r} & \dot{z} \end{bmatrix}^T, \tag{1}$$

and their derivatives are

$$\dot{u} = \begin{bmatrix} \ddot{\theta}_1 & \ddot{r} & \ddot{z} \end{bmatrix}^T. \tag{2}$$

The Cartesian boom-tip accelerations in cylindrical coordinates are

$$\ddot{x}_{tip} = (\ddot{r} - r\dot{\theta}_1^2)\cos\theta_1 - (r\ddot{\theta}_1 + 2\dot{r}\dot{\theta}_1)\sin\theta_1, \tag{3}$$

$$\ddot{y}_{tip} = (\ddot{r} - r\dot{\theta}_1^2)\sin\theta_1 + (r\ddot{\theta}_1 + 2\dot{r}\dot{\theta}_1)\cos\theta_1, \tag{4}$$

$$\ddot{z}_{tip} = \ddot{z}. \tag{5}$$

After substituting these relations into the sway dynamics, the θ_5 equation can be written in the input-acceleration-affine form

$$\ddot{\theta}_5 = \alpha_5\ddot{\theta}_1 + \alpha_6\ddot{r} + \alpha_7\ddot{z} + \varphi_5(x,u), \tag{6}$$

where

$$\alpha_5 = -\frac{m_0 l_0 + m l_3}{I_5} r\cos\theta_5\cos\theta_1, \tag{7}$$

$$\alpha_6 = -\frac{m_0 l_0 + m l_3}{I_5}\cos\theta_5\sin\theta_1, \tag{8}$$

$$\alpha_7 = -\frac{m_0 l_0 + m l_3}{I_5}\sin\theta_5, \tag{9}$$

with

$$I_5 = m_0 l_0^2 + m l_3^2, \qquad l_3 = l_1 + l_2\cos\theta_6. \tag{10}$$

The acceleration-free part is

$$\begin{aligned}\varphi_5(x,u) =& \frac{m_0 l_0 + m l_3}{I_5}\cos\theta_5\left(r\dot{\theta}_1^2\sin\theta_1 - 2\dot{r}\dot{\theta}_1\cos\theta_1\right)\\ &-\frac{b_5}{I_5}\dot{\theta}_5 - \frac{m_0 l_0 + m l_3}{I_5} g\sin\theta_5 + \frac{2 m l_2 l_3}{I_5}\sin\theta_6\dot{\theta}_6\dot{\theta}_5.\end{aligned} \tag{11}$$

Similarly, the θ_6 equation can be written as

$$\ddot{\theta}_6 = \beta_5\ddot{\theta}_1 + \beta_6\ddot{r} + \beta_7\ddot{z} + \varphi_6(x,u), \tag{12}$$

where

$$\beta_5 = \frac{r}{l_2}\left(-\cos\theta_6 \sin\theta_1 + \sin\theta_5 \sin\theta_6 \cos\theta_1\right), \tag{13}$$

$$\beta_6 = \frac{1}{l_2}\left(\cos\theta_6 \cos\theta_1 + \sin\theta_5 \sin\theta_6 \sin\theta_1\right), \tag{14}$$

$$\beta_7 = -\frac{\cos\theta_5 \sin\theta_6}{l_2}. \tag{15}$$

The acceleration-free part is

$$\begin{aligned}\varphi_6(x,u) =& \frac{1}{l_2}\Bigg[- r\dot{\theta}_1^2 \left(\cos\theta_6 \cos\theta_1 + \sin\theta_5 \sin\theta_6 \sin\theta_1\right) \\ &+ 2\dot{r}\dot{\theta}_1 \left(-\cos\theta_6 \sin\theta_1 + \sin\theta_5 \sin\theta_6 \cos\theta_1\right)\Bigg] \\ &- \frac{b_6}{ml_2^2}\dot{\theta}_6 - \frac{l_3}{l_2^2}\sin\theta_6 \dot{\theta}_5^2 - \frac{g}{l_2}\cos\theta_5 \sin\theta_6.\end{aligned} \tag{16}$$

The transformed velocity states are defined as

$$x_2 = \dot{\theta}_5 - \alpha_5 u_1 - \alpha_6 u_2 - \alpha_7 u_3, \tag{17}$$

$$x_4 = \dot{\theta}_6 - \beta_5 u_1 - \beta_6 u_2 - \beta_7 u_3. \tag{18}$$

Differentiating x_2 gives

$$\dot{x}_2 = \ddot{\theta}_5 - \alpha_5 \dot{u}_1 - \alpha_6 \dot{u}_2 - \alpha_7 \dot{u}_3 - \dot{\alpha}_5 u_1 - \dot{\alpha}_6 u_2 - \dot{\alpha}_7 u_3. \tag{19}$$

Substituting the input-acceleration-affine form of $\ddot{\theta}_5$ yields

$$\dot{x}_2 = \varphi_5(x,u) - \dot{\alpha}_5 u_1 - \dot{\alpha}_6 u_2 - \dot{\alpha}_7 u_3. \tag{20}$$

Thus, all terms containing $\ddot{\theta}_1$, $\ddot{r}$, and $\ddot{z}$ cancel exactly.

Applying the same procedure to x_4 gives

$$\dot{x}_4 = \varphi_6(x,u) - \dot{\beta}_5 u_1 - \dot{\beta}_6 u_2 - \dot{\beta}_7 u_3. \tag{21}$$

Therefore, the transformed dynamics depend only on the state variables and the cylindrical velocity inputs, and the model can be written in the standard nonlinear state-space form

$$\dot{x} = f(x,u). \tag{22}$$

The cancellation can be independently verified by checking

$$\frac{\partial \varphi_5}{\partial \ddot{\theta}_1} = \frac{\partial \varphi_5}{\partial \ddot{r}} = \frac{\partial \varphi_5}{\partial \ddot{z}} = 0, \qquad \frac{\partial \varphi_6}{\partial \ddot{\theta}_1} = \frac{\partial \varphi_6}{\partial \ddot{r}} = \frac{\partial \varphi_6}{\partial \ddot{z}} = 0. \tag{23}$$

Hence, the transformation does not neglect the input-acceleration terms; it eliminates them analytically by construction.
